# DeepUbi: a deep learning framework for prediction of ubiquitination sites in proteins

**DOI:** 10.1186/s12859-019-2677-9

**Published:** 2019-02-18

**Authors:** Hongli Fu, Yingxi Yang, Xiaobo Wang, Hui Wang, Yan Xu

**Affiliations:** 1 Department of Information and Computing Science, University of Science and Technology Beijing, Beijing, 100083 China; 2 Institute of Computing Technology, Chinese Academy of Sciences, Beijing, 100190 China; 3 Beijing Key Laboratory for Magneto-photoelectrical Composite and Interface Science, University of Science and Technology Beijing, Beijing, 100083 China

**Keywords:** Ubiquitination, Deep learning, Convolutional neural networks

## Abstract

**Background:**

Protein ubiquitination occurs when the ubiquitin protein binds to a target protein residue of lysine (K), and it is an important regulator of many cellular functions, such as signal transduction, cell division, and immune reactions, in eukaryotes. Experimental and clinical studies have shown that ubiquitination plays a key role in several human diseases, and recent advances in proteomic technology have spurred interest in identifying ubiquitination sites. However, most current computing tools for predicting target sites are based on small-scale data and shallow machine learning algorithms.

**Results:**

As more experimentally validated ubiquitination sites emerge, we need to design a predictor that can identify lysine ubiquitination sites in large-scale proteome data. In this work, we propose a deep learning predictor, DeepUbi, based on convolutional neural networks. Four different features are adopted from the sequences and physicochemical properties. In a 10-fold cross validation, DeepUbi obtains an AUC (area under the Receiver Operating Characteristic curve) of 0.9, and the accuracy, sensitivity and specificity exceeded 85%. The more comprehensive indicator, MCC, reaches 0.78. We also develop a software package that can be freely downloaded from https://github.com/Sunmile/DeepUbi.

**Conclusion:**

Our results show that DeepUbi has excellent performance in predicting ubiquitination based on large data.

**Electronic supplementary material:**

The online version of this article (10.1186/s12859-019-2677-9) contains supplementary material, which is available to authorized users.

## Background

Ubiquitin was first discovered by Goldstein et al. in 1975 [1]. Ubiquitination, covalent attachment of ubiquitin to a variety of cellular proteins, is a common post-translational modification (PTM) in eukaryotic cells [2]. In the process of ubiquitination, ubiquitin is attached to substrates on lysine (K) residues by a three-stage enzymatic reaction. There are three enzymes involved-ubiquitin activating enzyme (E1s), ubiquitin conjugating enzyme (E2s) and ubiquitin ligating enzyme (E3s), which work one after another [3–5]. The ubiquitination system is responsible for many aspects of cellular molecular function, such as protein localization, metabolism, regulation and degradation [4–7]. It also participates in the regulation of various biological processes such as cell division and apoptosis, signal transduction, gene transcription, DNA repair and replication, intracellular transport and virus budding [4, 5]. Evidence has shown that ubiquitination has a close relationship with cell transformation, immune response and inflammatory response [8]. Abnormal ubiquitination status is also involved in many diseases. For example, the ubiquitination of metastasis suppressor 1, mediated by the skp1-cullin1-F- box beta-transducin repeat-containing protein, is essential for regulating cell proliferation and migration in breast and prostate cancers [9].

Due to the roles of ubiquitination, the precise prediction of ubiquitination sites is particularly important. Conventional experimental methods are time-consuming and labour-intensive, and thus computational methods are necessary as a supplementary approach [10, 11]. In recent years, a variety of machine learning methods have been applied to predict protein ubiquitination sites. Tung and Ho [12] developed a ubiquitination site predictor UbiPred, using support vector machine (SVM) with 31 informative physicochemical features selected from the published amino acid indices [13]. Radivojac [14] used a random forest algorithm to develop a predictor, UbPred, in which 586 sequence attributes were employed as the input feature vector. Zhao [15] adopted an ensemble approach to the voting mechanism. Lee [16] designed UbSite, which uses an efficient radial basis function (RBF) kernel to identify ubiquitination sites. Chen [17] proposed a predictor, CKSAAP_UbSite, using the composition of k-spaced amino acid pairs (CKSAAP). Cai [18] proposed a predictor utilizing the nearest neighbour algorithm. Chen [19] proposed a new tool, UbiProber, which was designed for general and specific species. Chen [20] developed hCKSAAP_UbSite by integrating four different types of predictive variables. Qiu [21] developed iubiq-lys using support vector machine. Cai and Jiang [22] used multiple machine learning algorithms to predict ubiquitination sites. Wang [23] designed a tool, ESA-UbiSite, using an evolutionary algorithm (ESA). In addition, there are many other predictors such as UbiSite [24], UbiBrowser [25], RUBI [26], the WPAAN classifier [27], MDDLogoclustered SVM models [28] and the non-canonical pathway network [29]. Although various ubiquitination site predictors have been developed, there are still limitations. As noted above, the existing computational methods for predicting ubiquitination sites are shallow machine learning methods and their datasets are small. However, a large amount of biomedical data has been accumulated and shallow machine learning algorithms do not handle big data well. In this study, we propose a lysine ubiquitination predictor, DeepUbi, using a deep learning framework on a large dataset.

## Results

### Cross-validation performance

For the series of hyperparameter choices, we obtain a set of better performing hyper-parameters, which are shown in Table 1. Using a set of clear and effective metrics defined in Eq. 4 to measure the quality of predictors, we considered how to objectively derive the values. Three different verification methods are generally used to evaluate the predictive performance: the independent dataset test, sub-sampling test and jackknife test [30]. The jackknife test can exclude the “memory” effect and the arbitrariness problem because the outcome obtained by the jackknife cross-validation is always unique for a given benchmark dataset [21]. However, it is time-consuming, especially for big datasets. In this study, k-fold cross validation was utilized to evaluate the performance of the proposed predictors because of the large dataset.

**Table 1:** The values of super-parameter tuning

Super-Parameter	Preferred Setting
Embedding length	21
Batch size	65
Maximum epoch	30
Convolution blocks	([2–6], 64, ReLU)
Fully connected layer units	128
Cutoff	0.5
Dropout	0.7
Learning rate	0.01 with decay rate 0.95
Regularization	L2

**Fig. 1:**
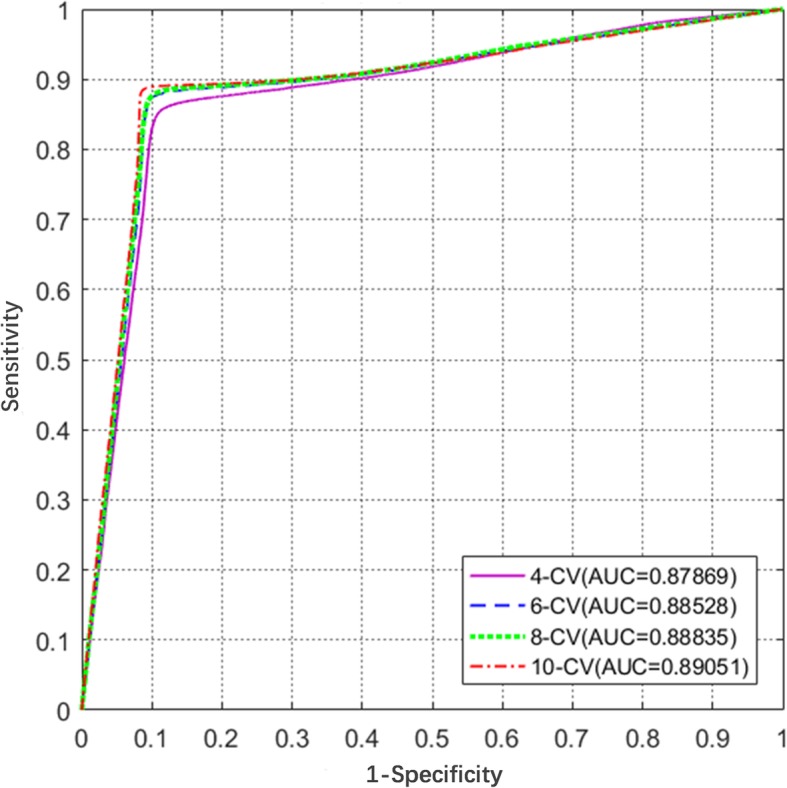
ROC curves of different cross-validations. ROC curves and their AUC values of 4-, 6-, 8-, and 10-fold cross validations with the One-Hot encoding scheme

**Fig. 2:**
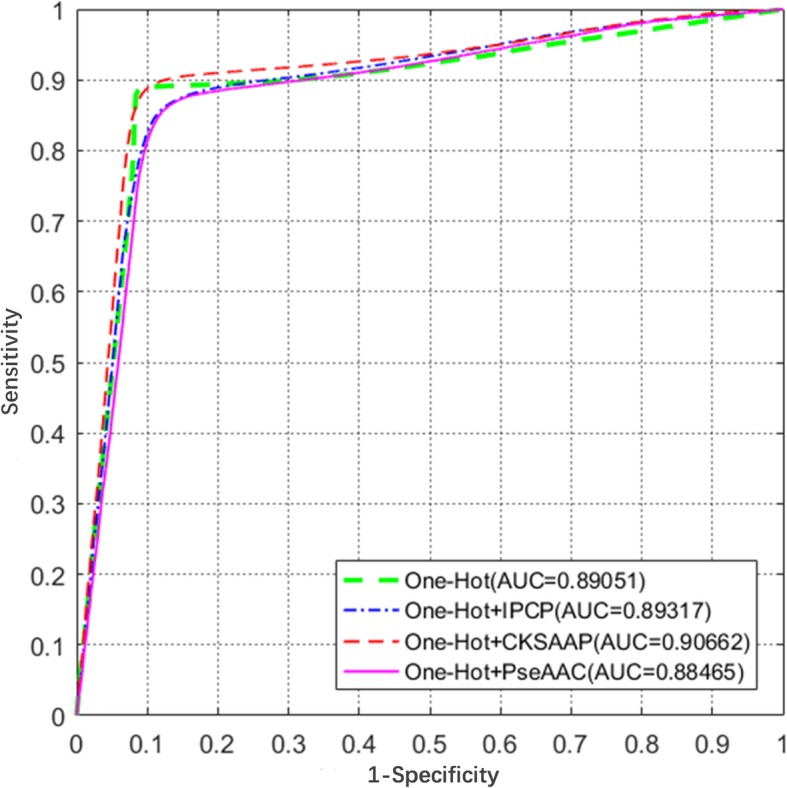
ROC curves of different feature constructions. ROC curves and their AUC values of four features in the 10-fold cross validation. These curves are very close to each other which illustrate the robustness of the model

First, the 4-fold, 6-fold, 8-fold and 10-fold cross validations are executed 10 times on the simple One-Hot encoding scheme. The results are shown in Table 2. All of the accuracies are greater than 85% and the highest accuracy reaches 88.74%, illustrating the robustness of the CNNUbi. The ROC curves and AUC values are shown in Fig. 1 and are more intuitive, and the largest AUC value was 0.89. These results show that the deep learning framework learns some instinct information and has good performance. To obtain more information, we add three other features into the One-Hot encoding scheme (see Table 3 and Fig. 2). In the 10-fold cross-validation, all the ROC curves are very close to each other. The One-Hot plus CKSAAP encoding scheme clearly performs the best in all of these features. We call it DeepUbi with an AUC of 0.9066 and MCC of 0.78.

**Table 2:** The results of 4-, 6-,8-,10-fold cross-validations with the One-Hot feature

Cross-Validation	*Acc* (%)	*Sn* (%)	*Sp* (%)	AUC	*MCC*
4-fold	86.86	84.33	89.57	0.8787	0.74
6-fold	88.47	86.65	90.43	0.8853	0.77
8-fold	88.06	88.26	87.84	0.8884	0.76
10-fold	89.58	87.65	91.65	0.8905	0.79

**Table 3:** The results of four different encoding schemes in the 10-fold cross-validation

Features	*Acc* (%)	*Sn* (%)	*Sp* (%)	AUC	*MCC*
One-Hot	89.58	87.65	91.65	0.8905	0.79
One-Hot + CKSAAP	88.98	89.80	88.10	0.9066	0.78
One-Hot + PseAAC	86.05	87.58	84.41	0.8847	0.72
One-Hot + IPCP	86.41	83.44	89.61	0.8932	0.73

Our DeepUbi predictor was obtained using balanced data. In the experimentally verified ubiquitination and non-ubiquitination data, the ratio of positive and negative peptides was 1:8. We also tested the performance on naturally distributed data when the algorithm was trained with balanced data. The results in Table 4 illustrate that the performance is slightly worse than with balanced data.

**Table 4:** The results for naturally distributed DeepUbi data

No. of fragments	Acc (%)	Sn (%)	Sp (%)	AUC	MCC	Pos:Neg
900	50.56	45.50	91.00	0.5490	0.23	1:8
9000	49.56	44.46	90.30	0.6626	0.22	1:8

### Comparison with other existing methods

A comprehensive comparison of our models with the available sequence-based predictors was performed and the corresponding data and results are shown in Table 5. In the last decade, many researchers have contributed to the prediction and research of ubiquitination sites in proteins. The comparison shows that the deep learning model performs very well on big datasets. The predictors improved the accuracy by adding new features, using a variety of machine learning algorithms or adding new datasets. The precision of the predictors is approximately 0.8. In this study, we propose the DeepUbi predictor and apply a deep learning framework with more accuracy. The AUC close to 0.9 and other indicators of accuracy, sensitivity and specificity are also better than those of existing methods. These results suggest that DeepUbi learned deeper characteristics.

**Table 5:** Comparison of DeepUbi and other ubiquitination prediction tools

Predictor	No. of positive samples	*Acc* (%)	*Sn* (%)	*Sp* (%)	AUC	*MCC*
UbiPred	151	84.44	83.44	85.43	0.85	0.69
UbPred	265	72.0	–	–	0.79	–
UbSite	385	74.5	65.5	74.8	–	–
CKSAAP_UbSite	263	73.4	69.85	76.96	0.81	0.47
UbiProber	22,192	–	37.0	90.0	0.77	0.63
hCKSAAP_UbSite	9537	–	–	–	0.77	–
iUbiq-Lys	659	82.14	80.56	99.39	–	0.50
ESA-UbiSite	85	94.0	96.0	92.0	–	0.92
DeepUbi	53,999	88.98	89.80	88.10	0.91	0.78

To eliminate the impact of data volume differences and make a more vivid comparison, we conduct additional experiments. We randomly select the same number of positive and negative samples as the existing predictor from our data 10 times. Each sample set is tested with 10 cross-validations, and the average results are listed in Table 6. Comparison of Table 5 and Table 6 shows that the DeepUbi results are much higher than those of other predictors for the same number of samples. For example, the data in UbiPred has an Acc of 84.44%, Sn of 83.44%, Sp of 85.43%, AUC of 0.85 and MCC of 0.69. Selecting the same number UbiPred data as the test set 10 times, the average result for DeepUbi is an Acc of 98.77%, Sn of 98.87%, Sp of 98.67%, AUC of 0.99 and MCC of 0.98. The AUC values of DeepUbi are close to 0.9, illustrating the performance of deep learning.

**Table 6:** The DeepUbi results for the same number of samples as the other existing tools

No. of positive samples	*Acc* (%)	*Sn* (%)	*Sp* (%)	AUC	*MCC*
UbiPred	84.44	83.44	85.43	0.85	0.69
DeepUbi	98.77	98.87	98.67	0.9993	0.98
UbPred	72.0	–	–	0.79	–
DeepUbi	98.51	98.45	98.57	0.9975	0.97
UbSite	74.5	65.5	74.8	–	–
DeepUbi	97.99	97.79	98.18	0.9933	0.96
CKSAAP_UbSite	73.4	69.85	76.96	0.81	0.47
DeepUbi	99.19	98.96	99.42	0.9959	0.98
UbiProber	–	37.0	90.0	0.77	0.63
DeepUbi	91.83	90.12	93.55	0.9093	0.84
hCKSAAP_UbSite	–	–	–	0.77	–
DeepUbi	94.10	92.31	95.89	0.9289	0.88
iUbiq-Lys	82.14	80.56	99.39	–	0.50
DeepUbi	98.92	98.90	98.93	0.9913	0.98
ESA-UbiSite	94.0	96.0	92.0	–	0.92
DeepUbi	95.59	95.53	95.65	0.9947	0.91

### Analysis of ubiquitination peptides

**Fig. 3:**
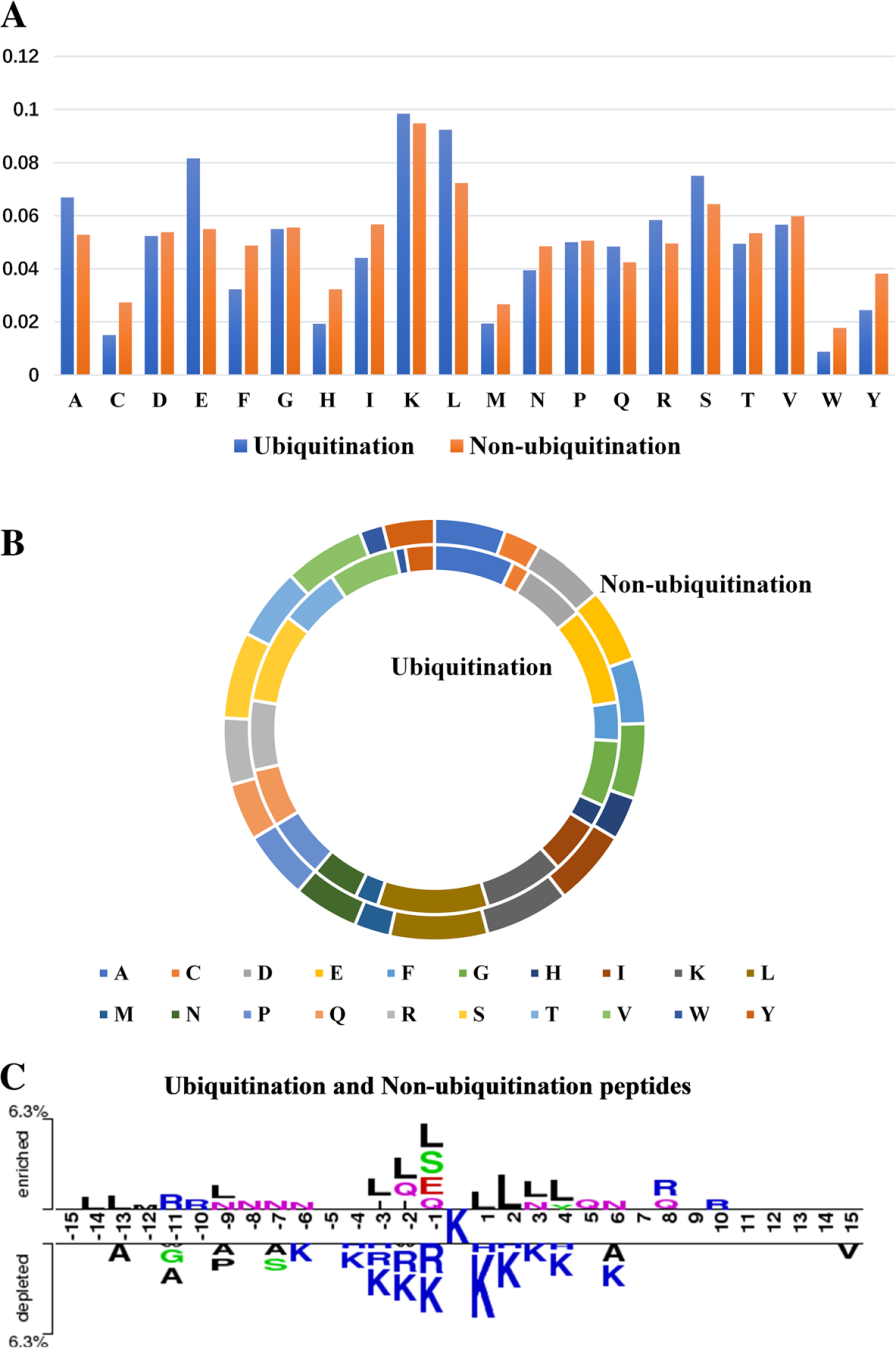
Different sequence analysis charts about ubiquitination and non-ubiquitination peptides. **a** A bar chart to compare the number of flanking amino acids surrounding the ubiquitination and non-ubiquitination peptides. **b** A circular chart to compare the percentage of flanking amino acids surrounding the ubiquitination and non-ubiquitination peptides. **c** Two Sample Logos web-server to calculate and visualize differences between ubiquitination and non-ubiquitination peptides

To illustrate the performance of our predictor, we also conduct an analysis using the training data. First, the probabilistic histogram of composition of flanking amino acids surrounding the ubiquitination candidate sites is generated, as shown in Fig. 3a and b. Amino acid residues Ala (A), Glu (E), Leu (L), Arg (R) and Ser (S) appear more ratio in positive data (ubiquitination fragments), while Cys (C), Phe (F), His (H), Ile (I) and Val (Y) are more enriched in negative data (non-ubiquitination fragments). Next, a well-known tool, Two Sample Logo [31], is applied to detect the position-specific amino acid composition difference between the training data, and the sequence logo is shown in Fig. 3c. The results reveal the dependencies of flanking amino acids around the substrate sites.

## Discussion

We use the biggest data repository designed for protein lysine modification to learn the DeepUbi predictor. A convolutional neural network, a deep learning framework, is adopted to predict ubiquitination. It is composed of a convolutional layer, a nonlinear layer and a pooling layer. Convolutional neural networks can learn a large number of mapping relations between input and output without any precise mathematical expression between the input and output. We construct six steps, including inputting the fragment, constructing an embedding layer, building multi-convolution-pooling layers, adding features, constructing fully connected layers, and the output layer. The deep learning framework is first used to predict ubiquitination.

Four better encoding schemes are adopted in the feature construction, One-Hot encoding, the physicochemical properties, the composition of k-spaced amino acid pairs (CKSAAP) and the pseudo amino acid composition. One-Hot plus CKSAAP have the best performance with and AUC of 0.9066 in the cross-validation.

In the data, the sequence motif analysis shows that there are differences between positive and negative fragments. Thus, it is feasible to obtain classification information from the peptide itself. Different features are adopted to train the model. The hybrid of One-Hot and CKSAAP is selected as the best, with an AUC of 0.9066.

DeepUbi has better performance than the existing tools. Researchers could use the predictor to select potential candidates and conduct experiments to verify them. This will reduce the range of candidate proteins and save time and labour. The sequence analysis of the ubiquitination will provide suggestions for future work.

In the future, we will investigate other feature constructions that may better extract the properties of samples. Second, we aim to improve performance by increasing the depth and model parameters through system learning. The current method may also be used to identify other PTM sites in proteins.

## Conclusion

In this work, we propose a new ubiquitination predictor, DeepUbi, which uses a deep learning framework and achieves satisfactory success with the biggest data set. DeepUbi extracts features from the original protein fragments with an AUC of 0.9066 and an MCC of 0.78. We construct six steps including inputting fragment, constructing an embedding layer, building multi-convolution-pooling layers, adding features, constructing fully connected layers, and output layer. The deep learning framework is first used in prediction of ubiquitination. However, DeepUbi is not too deep, as we only use two convolution-pooling structures. We also develop a software package for DeepUbi that can be freely downloaded from https://github.com/Sunmile/DeepUbi. The deep learning model is an effective prediction method and will improve accuracy by increasing the depth in the future.

## Methods

### Benchmark dataset

**Fig. 4:**
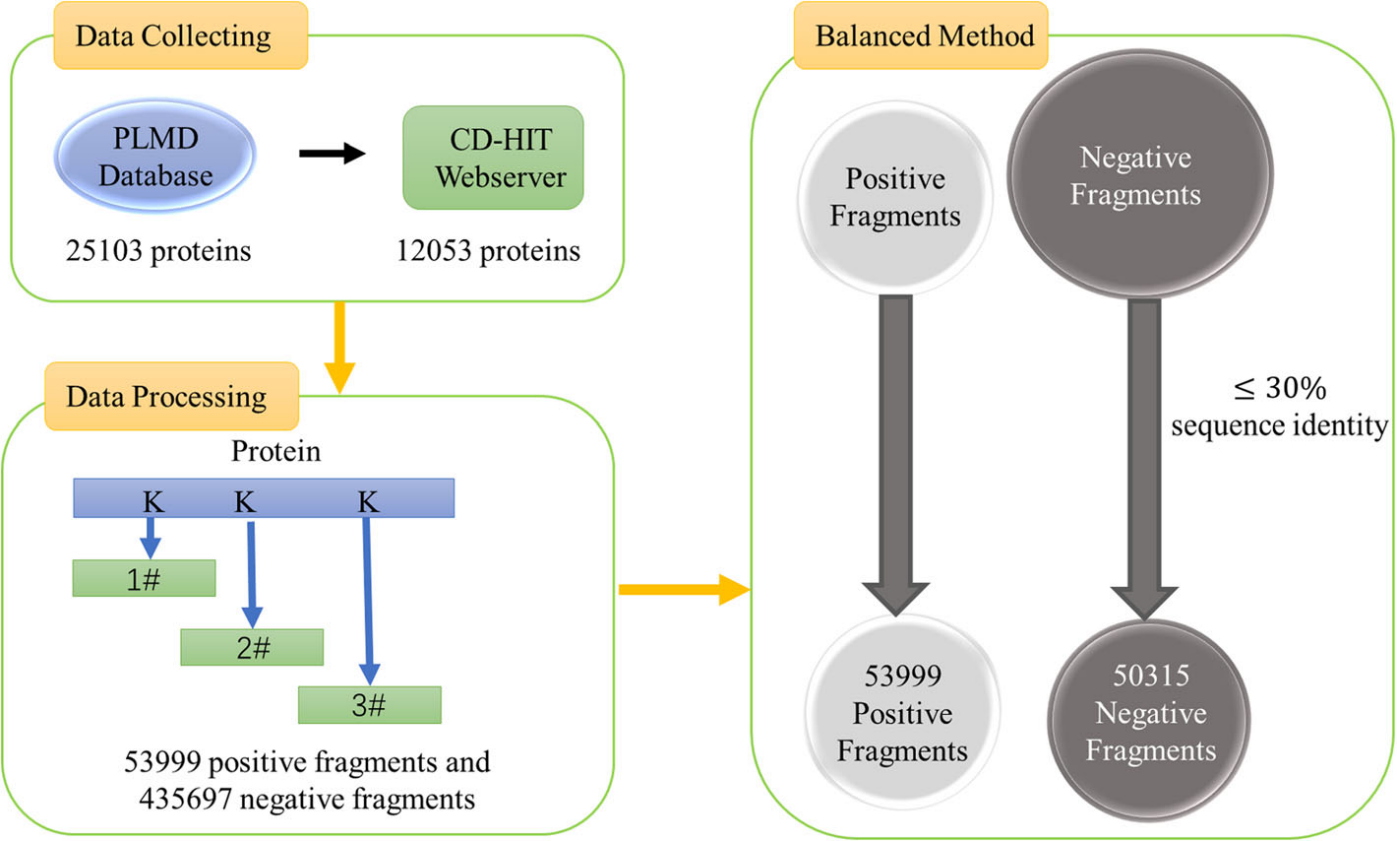
Flow chart of the data collection and processing. Firstly, collecting the raw proteins and then removing the redundant protein sequences with CD-Hit; secondly, intercepting the protein sequences with a 31 sliding window to get the positive and negative fragments; at last, using 30% identity in negative samples to get a balanced training data

In this study, the ubiquitination data is collected from the PLMD (v3.0, June. 2017) database [32], which is the biggest online data repository designed for protein lysine modification. The original data contains 121,742 ubiquitination sites from 25,103 proteins. If the data contains homologous samples, it would increase the bias of results. We remove the redundant protein sequences to eliminate homology bias using the CD-HIT web server [33], which is freely available at http://weizhongli-lab.org/cd-hit/, and obtains 12,053 different proteins with ≤30% sequence identity. A sliding window with the length of 15 × 2 + 1 = 31 is used to intercept the protein sequences with lysine residues in the centre. If the upstream or downstream residues of a protein are less than 15, the lacking residue is filled with a “pseudo” residue ‘X’. There are too many negative peptides compared to the positive peptides. To obtain a better predictor, we select the negative samples by deleting the redundant segments using 30% identity to ensure that none of the segments had ≥30% pair-wise identity in the negative peptides [24]. Finally, we obtain a training dataset containing 53,999 ubiquitination and 50,315 non-ubiquitination fragments. A detailed flow chart of these steps is shown in Fig. 4.

### Feature construction

A good feature can extract the correlation of instinct ubiquitination characters and the targets from peptide sequences [34]. Four better feature encoding schemes are adopted, One-Hot encoding, the physicochemical properties, the composition of k-spaced amino acid pairs and the pseudo amino acid composition.

One-Hot Encoding.

The conventional feature representation of amino acid composition uses 20 binary bits to represent an amino acid. To deal with the problem of sliding windows spanning out of the N-terminal or C-terminal, one additional bit is appended to indicate this situation. Then, a vector of size (20 + 1) bits is used to represent a sample. For example, the amino acid A is represented by ‘100000000000000000000’ and R is represented by ‘010000000000000000000’.

#### Informative physicochemical properties (IPCP)

In PTM site prediction, physicochemical properties are essential to extract information for a fragment or protein. Tung [12] proposed an informative physicochemical property mining algorithm that could quantify the effectiveness of individual physicochemical properties in prediction. They used the value of the main effect difference (MED) [35] to estimate the individual effects of physicochemical properties. The property with the largest MED is the most effective in predicting ubiquitination sites. In the study, 31 informative physicochemical properties are selected as the features for calculation, and are listed in Additional file 1: Table S1.

#### Compositions of *K*-spaced amino acid pairs (CKSAAP)

The CKSAAP encoding scheme is the composition of *k*-spaced residue pairs (separated by *k* amino acids) in the protein sequence, which is useful for predicting protein flexible or rigid regions [36]. For example, there are 441 residue pairs (i.e., AA, AC, ..., XX). Therefore, the feature vector can be defined as

1 $$ \left\{\frac{N_{AA}}{N_{to tal}}\kern0.75em \begin{array}{cc}\frac{N_{AC}}{N_{to\mathrm{t} al}}&, \end{array}\cdots, \kern0.5em \begin{array}{cc}\frac{N_{XX}}{N_{to tal}}&\ \end{array}\right\} $$

where *N*_*total*_ is the total number of *k*-spaced residue pairs in the fragment and *N*_*AA*_ is the number of amino acid pair AA in the fragment. Each component in the vector represents the contribution of *k*-spaced amino acid pairs. For instance, the AA component is represented as $\frac{N_{AA}}{N_{total}}$. In this paper, *k* = 0, 1, 2, 3, 4, and a 441 × 5 = 2205 vector was obtained by the CKSAAP encoding scheme.

Pseudo Amino Acid Composition (PseAAC).

Chou’s pseudo amino acid composition is a set of discrete serial correlation factors combined with traditional 20 amino acid components [37]. In the study, we select 20 correlation factors and the weight of these factors is 0.05, and a 40-dimension vector is acquired.

### Algorithm

**Fig. 5:**
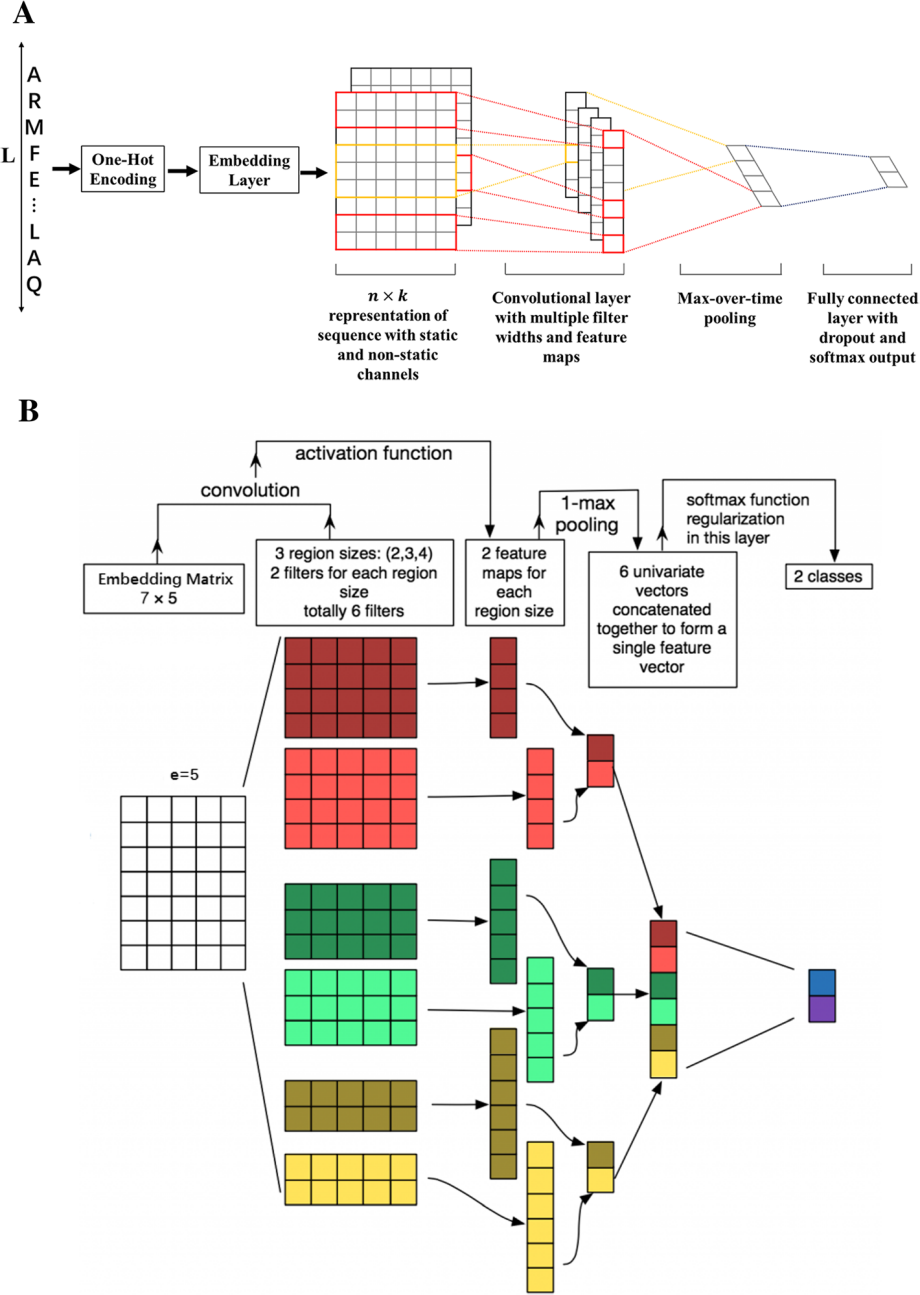
**a** Flow chart of the CNN deep learning model. **b** An example of convolution-pooling structure. **a** Input a fragment and encode; construct an embedding layer; build multi-convolution-pooling layers; construct fully connected layers; and then get the output. **b** Use different filters with different sizes to get a series of feature maps; and then use a max-pooling and concatenating together to form a feature vector. Finally, the softmax function regularization is used to get the classification

Deep learning, which evolved from the acquisition of big data, and the power of parallel and distributed computing have facilitated major advances in numerous domains such as image recognition, speech recognition, and natural language processing [38]. Every protein is a sentence, and residues in the protein sequence can be seen as “words”. The prediction of ubiquitination can be seen as a ‘natural language prediction’ (NLP) task. Therefore, we propose a convolutional neural network (CNN) deep learning model and obtain good prediction performance on a large data set. A convolutional neural network (CNN) is a deep learning framework. It is composed of a convolutional layer, a nonlinear layer and a pooling layer. Our model is constructed with six steps (Input a fragment, Construct an embedding layer, Build multi-convolution-pooling layers, Add features, Construct fully connected layers, and an Output layer), as shown in Fig. 5a.

The input protein fragment representation is *x* ∈ *R*^*L* × 21^, where *L* is the length of the fragment. The first layer is the embedding layer, which maps input vectors into low-dimensional vector representations. It is essentially a lookup table that we learn from data. E = *xW*_*e*_, where *e* is the embedding dimension, *W*_*e*_ is the embedding weight matrix and E ∈ *R*^*L* × *e*^ is the embedding matrix, which is a continuous product. Then, we assign the embedding matrix E as an image and use the convolutional neural network to extract features. Because the adjacent residues in the fragments are always highly correlated, one dimensional convolution can be used. The width of the convolution kernel is the dimension of the embedding vector. The height is a super parameter, which is a manual set. For example, if there is a convolution filter with size *a*_*k*_, then a feature map is obtained by the convolution

2 $$ {\mathrm{z}}_k(m)=f\left({\sum}_{i=1}^{a_k}{\sum}_{j=1}^ew\left(i,j\right)\times E\left(i+m,j\right)\right) $$

where *f* is the activation function, which is a rectified linear unit (ReLU) [39], *w* is the weight vector and ${\mathrm{z}}_k\in {R}^{L-{a}_k+1}$. The number of convolution filters of size *a*_*k*_ is also set. The feature map obtained from different convolution kernels is a different size, so a max-pooling function is use to maintain the same dimension. The final eigenvector *h* is then obtained. For more intuitive understanding, see Fig. 5b. For the first model, CNNUbi, we use the features obtained from the last step without additional features, i.e., *h*_*new*_ = *h*. For comparison, the second model, DeepUbi, is built with additional features and *h*_*new*_ = [*h*, *b*], where *b* is the additional features. Finally, each of the two output units has a score between 0 and 1, illustrating by the softmax equation ${p}_i=\frac{e^i}{\sum_j{e}^j}$. Here, *i* = *F*_*c*_*w*_*o*_ represents the input of class unit *i*, *F*_*c*_ is the output of the fully connected layer and *w*_*o*_ is the weight matrix. The cross-entropy objective function is assigned as the cost function Add features

3 $$ \mathrm{CE}=-{\sum}_{n=1}^N{y}^n\ln P\left({y}^n=1|{x}^n\right)+\left(1-{y}^n\right)\ln P\left({y}^n=0|{x}^n\right) $$

where *N* represents the batch size of the training set and *x*^*n*^ and *y*^*n*^ represent the *n*-th protein fragment and its label, respectively. Using the Adam optimizers, DeepUbi is trained based on a variety of super-parameters such as the batch size, maximum epoch, learning rate, dropout rate and convolution blocks.

### Model evaluation and performance measures

A confusion matrix is a visual display tool for evaluating the quality of classification models. Each column of the matrix represents the sample situation of the model prediction and each row of the matrix represents the actual situation of the sample. There are four values in the matrix, where TP represents the number of true positives, TN is the number of true negatives, FP is the number of false positives, and FN is the number of false negatives. In the literature, the following metrics based on the confusion matrix are often used to evaluate the performance of a predictor

4 $$ \left\{\begin{array}{c}\begin{array}{c}\mathrm{Sp}=\frac{TN}{TN+ FP}\\ {}\mathrm{Sn}=\frac{TP}{FN+ TP}\end{array}\ \\ {}\begin{array}{c}\mathrm{Acc}=\frac{TP+ TN}{TP+ TN+ FP+ FN}\ \\ {}\mathrm{MCC}=\frac{TP\times TN- FP\times FN}{\sqrt{\left( TP+ FN\right)\left( TN+ FP\right)\left( TP+ FP\right)\left( TN+ FN\right)}}\end{array}\end{array}\ \right. $$

where *Sn* represents the sensitivity, *Sp* is the specificity, *Acc* is the accuracy, and *MCC* is the Matthew’s correlation coefficient. The ROC (Receiver Operating Characteristic) curves and the area under the ROC curve (AUC) are usually used to evaluate the classifier’s resolving power.

## Additional file

Additional file 1: **Table S1**. The 31 informative physicochemical properties and their corresponding MED (main effect difference) scores. (XLSX 42 kb)

## Abbreviations

Acc: Accuracy
AUC: Area under the ROC curve
CKSAAP: Composition of k-spaced amino acid pairs
CNN: Convolutional neural network
IPCP: Informative physicochemical properties
MCC: Mathew’s correlation coefficient
MED: Main effect difference
PseAAC: Pseudo amino acid composition
PTM: Post-translational modification
RBF: Radial basis function
ReLU: Rectified linear unit
Sn: Sensitivity
Sp: Specificity
SVM: Support vector machine
